# Editorial: Exploring the influence of gut microbiome on human health: mechanistic insights from pig models

**DOI:** 10.3389/fmicb.2025.1570451

**Published:** 2025-03-28

**Authors:** François Meurens, Shimeng Huan, Wen-Chao Liu, Lifeng Zhu, Wei Liu

**Affiliations:** ^1^Research Group on Infectious Diseases in Production Animals (GREMIP) & Swine and Poultry Infectious Diseases Research Center (CRIPA), Faculty of Veterinary Medicine, University of Montreal, St-Hyacinthe, QC, Canada; ^2^Department of Veterinary Microbiology and Immunology, Western College of Veterinary Medicine, University of Saskatchewan, Saskatoon, SK, Canada; ^3^State Key Laboratory of Animal Nutrition, China Agricultural University, Beijing, China; ^4^Department of Animal Science, College of Coastal Agricultural Sciences, Guangdong Ocean University, Zhanjiang, China; ^5^School of Medicine & Holistic Integrative Medicine, Nanjing University of Chinese Medicine, Nanjing, China; ^6^Plant Protection and Microbiology Research Institute, Zhejiang Academy of Agricultural Sciences, Hangzhou, Zhejiang, China

**Keywords:** pig, microbiota, gut, health, human health

The exploration of gut health in swine (Hu et al., 2024; Upadhaya and Kim, 2022), in humans (Hou et al., 2022) and in various species has gained considerable attention lastly, with multiple studies elucidating the intricate interplay between the gut microbiome, host genetics, and health outcomes. This editorial synthesizes findings from recent research in Frontiers in Microbiology that highlights the potential for dietary interventions and genetic considerations in enhancing swine health and productivity. It also presents two recent studies, in humans, where the gut microbiota has been shown as having a major impact.

In their study, Han et al. presented critical findings regarding the adverse effects of artificial piglet rearing on the intestinal microbiota and overall gut health. Through their study, they nicely demonstrated that artificially reared piglets experienced increased diarrhea incidence and detrimental alterations in gut microbiota composition. These outcomes raise essential questions about current management practices in pig production, underlining the necessity for strategies that mitigate the negative impacts of artificial rearing methods. Complementing that study, Jiang et al. examined an interesting relationship between testosterone levels and fecal microbiota in developing Meishan male pigs. Their research revealed dynamic interactions indicating that testosterone influences the composition of gut microbiota, while the microbiome may also play a role in testosterone metabolism. Understanding these relationships could serve as a foundation for developing breeding strategies aimed at optimizing hormonal health in pigs. Then, in an interesting and recent study, Liu W. et al. explored the effects of a multispecies probiotic complex on bile acid metabolism and gut microbiota using an *in vitro* fermentation model. They demonstrated significant alterations in bile acid profiles and modifications to gut microbiota composition following probiotic treatment. This indicates a promising therapeutic application of probiotic interventions for managing metabolic disorders and enhancing gut health in swine. Complementing the research on probiotics, Zhu et al. investigated the effects of maternal diets supplemented with probiotics and synbiotics on the colonic microbiome and metabolome of offspring. Their findings illustrated that maternal supplementation significantly shapes the gut microbiota composition in piglets, thereby emphasizing the critical role of maternal nutrition on progeny health. This study advocates for strategic maternal dietary interventions not only to enhance immediate gut health in offspring but also to promote long-term benefits for growth and disease resistance in swine. Zhu et al. argue for the implementation of probiotics during gestation and lactation as a means to promote a favorable microbiome in neonates, which could result in better health outcomes and productivity throughout the life of the pig. More specifically, Liu X. et al. focused on the probiotic bacterium *Clostridium butyricum* CBX 2021 strain, assessing its mechanisms in improving pig health through *in vivo* and *in vitro* approaches. Their study reported that strain CBX 2021 enhanced growth indicators and reduced diarrhea rates in weaned piglets. The probiotic also exhibited beneficial effects on immune response and gut microbiota composition, highlighting its potential role in developing new probiotic products to improve swine health. Still about the effect of one bacterium, Yuan et al. examined *Bacillus amyloliquefaciens* SC06's effects on weaned piglets experiencing endoplasmic reticulum (ER) and oxidative stress. The study demonstrated that SC06 supplementation significantly improved growth performance while decreasing markers of oxidative and ER stress. Through transcriptomic and metagenomic analyses, SC06 was shown to positively influence gene expression linked to antioxidant activity and to alter gut microbiome composition, thus contributing to better intestinal health in stressed piglets.

Regarding the use of amino acids, Liu G. et al. investigated the effects of tryptophan (Trp) supplementation in lipopolysaccharide (LPS)-stimulated piglets. Their findings revealed that Trp enhances the abundance of beneficial gut bacteria and short-chain fatty acids (SCFAs) while reducing inflammation and improving intestinal barrier function. This suggests that dietary Trp supplementation could serve as an effective strategy for mitigating the adverse effects of inflammatory challenges in piglets. On their side, Yu et al. investigated the effects of dietary galacto-oligosaccharides (GOS) and hyocholic acids (HCA) on sow reproductive performance, particularly during late gestation and lactation. Their study, involving 60 multiparous sows divided into four dietary groups, revealed that GOS and HCA supplementation significantly contributed to shorter labor times and increased piglet birth weights. Furthermore, they observed improvements in serum triglyceride levels and total antioxidant capacity among sows on supplemented diets. Notably, GOS and HCA also induced changes in gut microbiota composition, suggesting that dietary interventions could augment reproductive performance and enhance milk quality. These findings advocate for the strategic use of dietary nutrients in optimizing both sow health and offspring vitality, illustrating the cascading benefits of microbial modulation on reproductive success.

Focusing on the impact of the microbiota on distant organs, Qi et al. advanced our understanding of how gut microbiota influences hypothalamic function and overall health. Their whole transcriptome analysis illustrated significant alterations in gene expression related to energy metabolism and cell signaling in the presence or absence of gut microbiota. This interplay marks a crucial step in unraveling the microbiota-gut-brain axis, with important implications for metabolic and neurodegenerative diseases, emphasizing potential therapeutic interventions. Still in the field of microbio-endocrinology, Zheng et al. explored the interplay between intestinal melatonin levels and gut microbiota, focusing on how melatonin's synthesis occurs locally in the gut independent of the pineal gland's influence. Their research utilized a pig model with pinealectomy to investigate these effects. They found that while the removal of the pineal gland did not alter gastrointestinal melatonin levels or overall gut microbiota composition, supplementing melatonin led to significant changes in the gut microbiota structure. This indicates that melatonin synthesized in the intestinal tract may play a crucial role in regulating gut microbiota, reinforcing its importance in maintaining gut health and homeostasis. The findings underscore that melatonin might serve as a modifiable dietary component that can support gut health through its regulatory effects, presenting further opportunities for dietary supplementation in livestock management. To gain a better understanding of the causes of obesity, Zeng et al. studied fluctuations in the fecal microbiota of Bama minipigs during periods of weight gain and loss, highlighting the crucial role of gut microbiota in managing obesity. Their research demonstrated significant correlations between microbial diversity and variations in body weight, indicating that specific microbial communities may be associated with weight gain or loss. Zeng et al.'s findings suggest that targeted dietary or microbial interventions could serve as effective strategies for managing obesity not only in swine but potentially in human models as well. This study highlights the intricate relationships between diet, microbiota, and body weight regulation, paving the way for innovative approaches to tackle obesity through microbiome manipulation.

In elderly pigs, Qiao et al. investigated the relationship between the oral-gut microbiome and inflamm-aging employing a multi-omics approach. Their findings demonstrated age-dependent shifts in microbial composition correlating with systemic inflammation markers. These results underscore the relevance of gut health in aging, suggesting that targeted interventions could help manage gut microbiota and potentially extend healthy lifespan. Then, Wang et al. contributed to our understanding of the relationship between host genetics and gut microbiome composition regarding fat deposition traits. By analyzing a resource population of pigs, they identified specific microbial candidates correlated with the percentage of leaf fat and intramuscular fat content. This research emphasizes the significance of microbiome interactions in optimizing meat quality and refining selection criteria for future pig breeding strategies.

In a review article, Yang et al. examined the role of gut archaea within the swine microbiome. They highlighted the previously understudied diversity of gut archaea and their potential links to economically important traits, such as weight gain and feed efficiency in pigs. While bacteria have traditionally received most of research attention, Yang et al. advocated for a more inclusive approach that investigates archaeal species. The mini-review discusses how these microorganisms could act as potential probiotics, enhancing pig growth and overall health. They suggest that further research into the metabolic functions of gut archaea could uncover unique contributions to the gut environment that support digestion, nutrient absorption, and immune function in swine. Thus, integrating archaeal research into swine breeding and health management could lead to innovative strategies for improving livestock productivity and resilience.

In their study in humans, Chen et al. investigated the causal relationship between gut microbiota and leukemia using a two-sample Mendelian randomization method. They identified ten gut microbial taxa associated with leukemia risk. Notably, *Blautia* and *Lactococcus* were recognized as risk factors for acute lymphoblastic leukemia, while several *Ruminococcaceae* taxa were associated with chronic myeloid leukemia. This study highlights the potential of gut microbiota as biomarkers for leukemia, suggesting that microbiome modulation could offer new avenues for early detection and targeted treatment strategies. Still in the human species, Fu and Hao explored the causal relationships between gut microbiota (GM) and non-Hodgkin lymphoma (NHL) using bidirectional Mendelian randomization analysis. Their findings identified 27 specific gut microbial species linked to NHL, with 20 showing a negative association and seven a positive one. This research emphasizes the complex role of gut microbiota in cancer development, indicating that understanding these relationships could lead to innovative preventive and therapeutic strategies for NHL.

Collectively, these studies, in pigs, pig biomedical model, and humans, offer profound insights into the multifaceted relationships between dietary interventions, microbiota composition, and overall health. They inform future strategies aimed at enhancing animal welfare and productivity through targeted microbiome management, dietary enrichment, and innovative breeding practices. The findings across these diverse studies illustrate the importance of integrating gut health considerations into all aspects of swine production, from gestation through to market readiness.
